# Supplementary material for: Integrating phylogeography and high-resolution X-ray CT reveals five new cryptic species and multiple hybrid zones among Australian earless dragons
Source: R Soc Open Sci. 2019 Dec 18;6(12):191166. doi: 10.1098/rsos.191166 (PMC6936289; doi:10.1098/rsos.191166)
Supplement: FIGURE APPENDIX S2 [file rsos191166supp3.pdf]

FIGURE APPENDIX S2

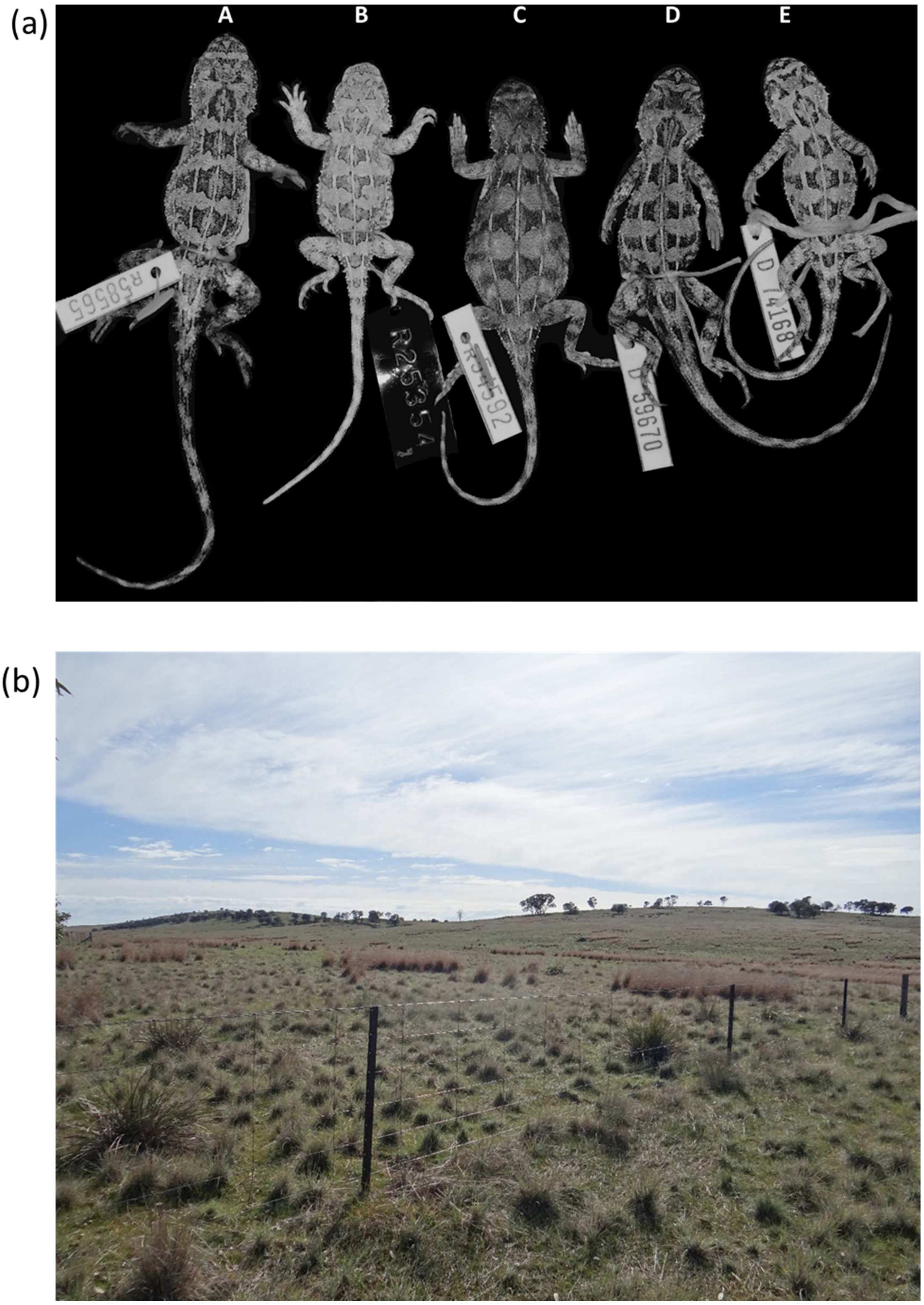

Figure S1. *Tympanocryptis petersi* sp. nov.: (a) species variation (A, SAMA R58565, male, Eyre Peninsula; B, SAMA R25354, male, Lake Gilles, South Australia; C, SAMA R54592, female, Blanchetown, South Australia; D, NMV D59670, Hattah, Victoria; E, NMV D74168, male, Lake Tyrell, Victoria); and (b) grassland habitat north of Jamestown, South Australia.

(a) (b)

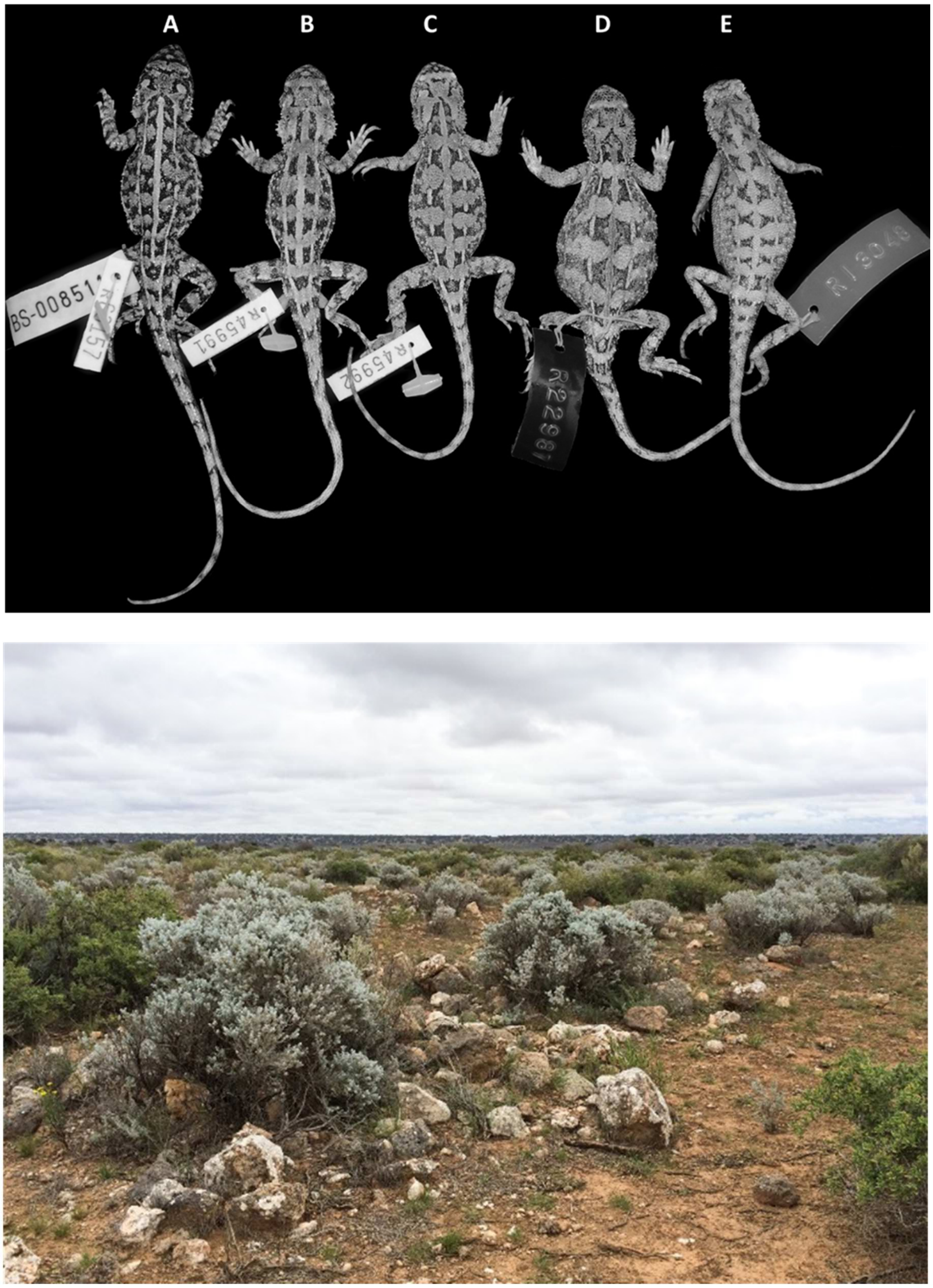

Figure S2. *Tympanocryptis houstoni:* (a) variation within species (A, SAMA R63157, male, Nullarbor Plain, South Australia; B, SAMA R45991, male, Cook, South Australia; C, SAMA R45992, female, Cook, South Australia; D, SAMA R22987, Cocklebiddy, Western Australia; E, SAMA R13048, Nullarbor Station, South Australia.); and (b) habitat, Nullarbor, Western Australia (S. Zozaya).

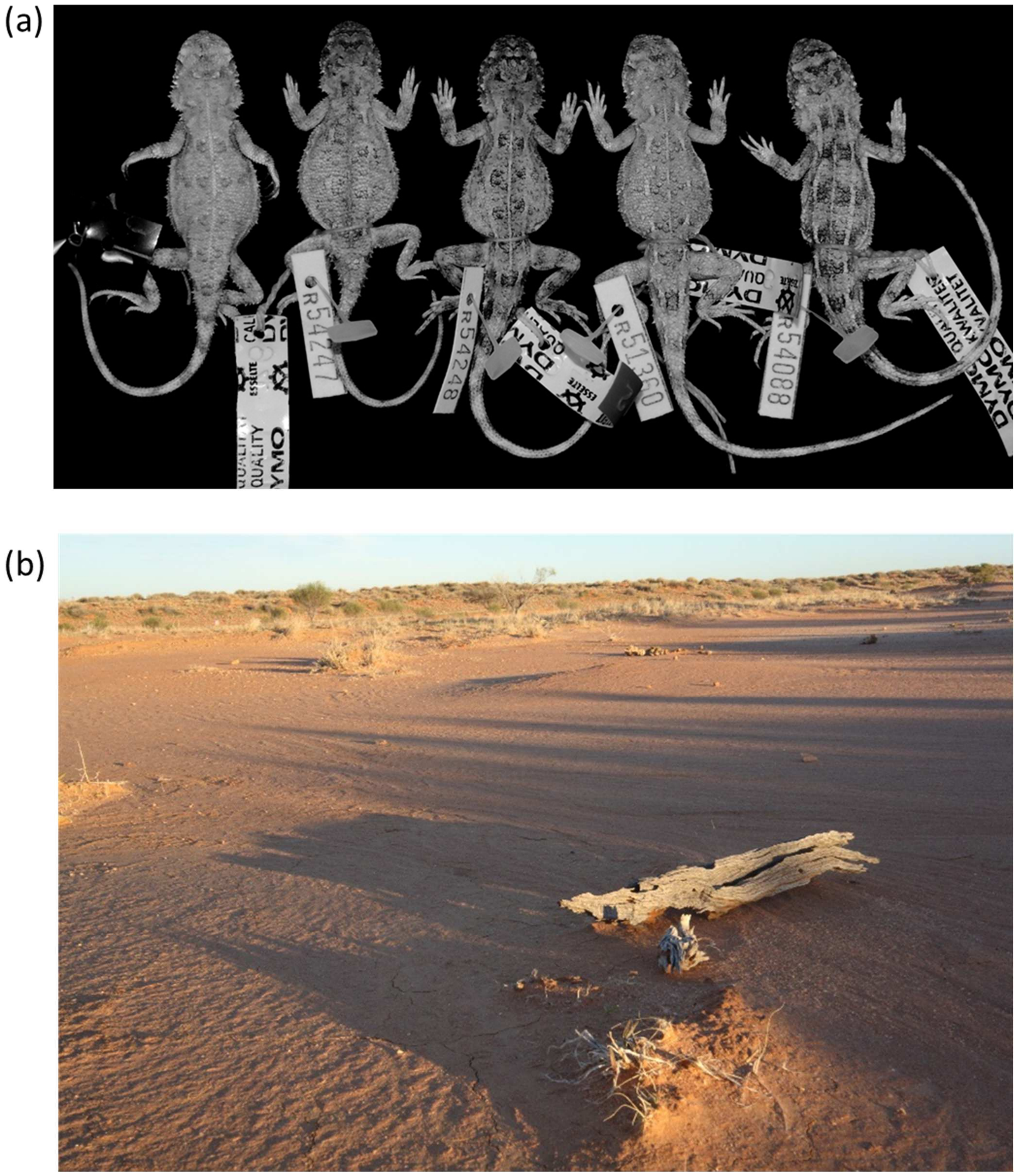

Figure S3. *Tympanocryptis argillosa sp.* nov: (a) variation within species (A, SAMA R03619, female, Lake Palankarinna ; B, SAMA R54247, female, Kalamurina ; C, SAMA R54248, male, Kalamurina; D, SAMA R51360, male, Simpson Desert ; E, SAMA R54088, male, Kalamurina); and (b) habitat, Innamincka area, South Australia (S. Wilson).

(a)
(b)

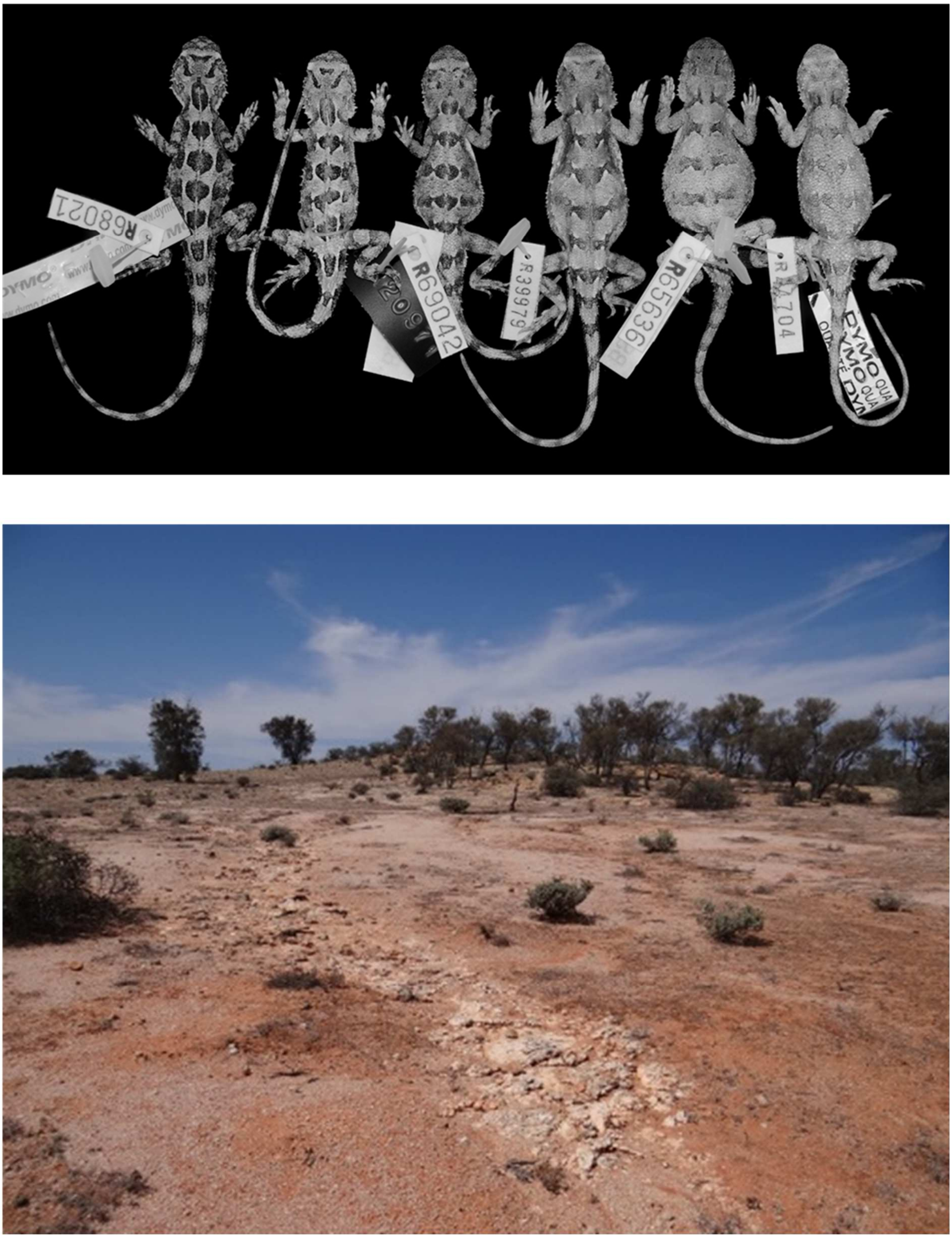

Figure S4. *Tympanocryptis tolleyi sp. nov.:* (a) variation within species (A, SAMA R68021, male, Tarcoola; B, SAMA R20971, male, Roxby Downs; C, SAMA R69042, male, Mt Igy, South Australia; D, SAMA R39979, female, Bulgunnia Station; E, SAMA R65636, female, The Twins Station; F, SAMA R44704, female, Bon Bon Station); and (b) habitat, east of Kychering Rocks, South Australia (M. Hutchinson).

(a)

(b)

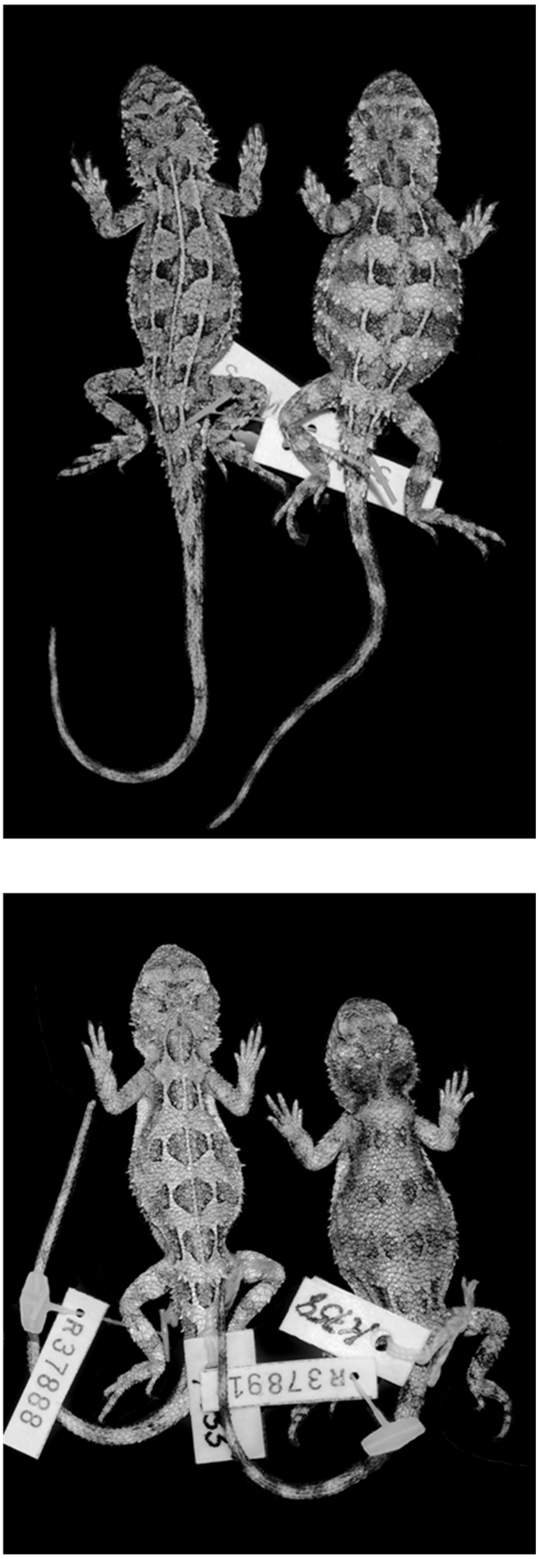

Figure S5. Specimens from *Tympanocryptis tolleyi sp. nov.* contact zones: (a) Specimens from the eastern Gawler Ranges, 93 km NE of Minnipa, showing typical male (left) and female (right) colour patterns intermediate between *T. tolleyi* and *T. petersi*.; and (b) A male (left) and female (right) *T. tolleyi f*rom the Olympic Dam region, South Australia - although showing the typical external morphology of *T. tolleyi*, these specimens carry the mtDNA typical of *T. argillosa*. The male (SAMA R37888) was also sequenced for Rag-1 and fell out with *T. tolleyi*, not *T. argillosa*.

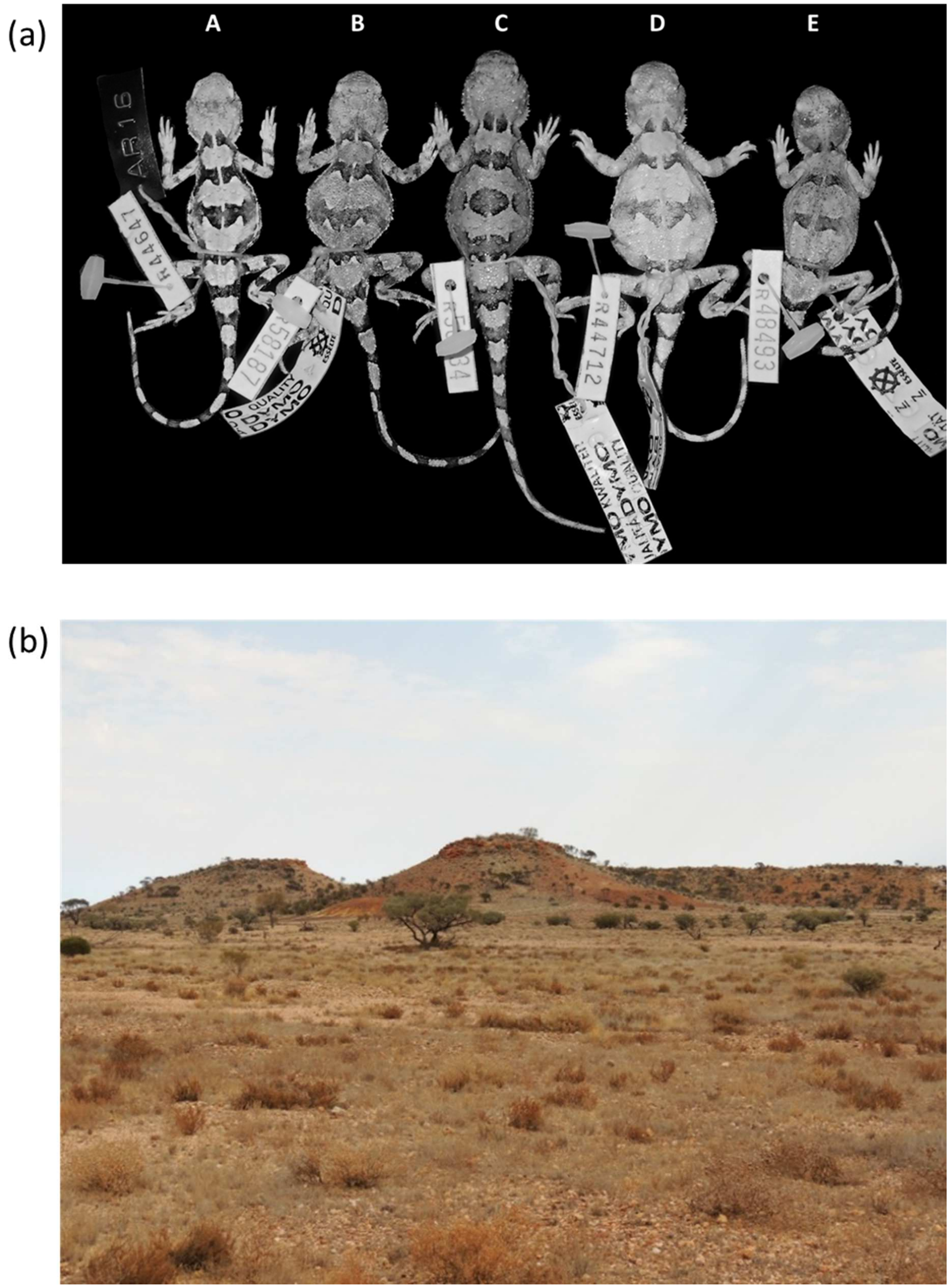

Figure S6. *Tympanocryptis fictilis sp. nov.*: (a) variation within species (A, SAMA R44647, male, Todmorden Station; B, SAMA R58187, male, Pile Hill, The Breakaways; C, SAMA R58134, male, England Hill; D, SAMA R44712, female, Todmorden Station; E, SAMA R48493, female, Todmeroden Station); and (b) habitat, Copper hills Station, South Australia (M. Hutchinson).

(a)
(b)

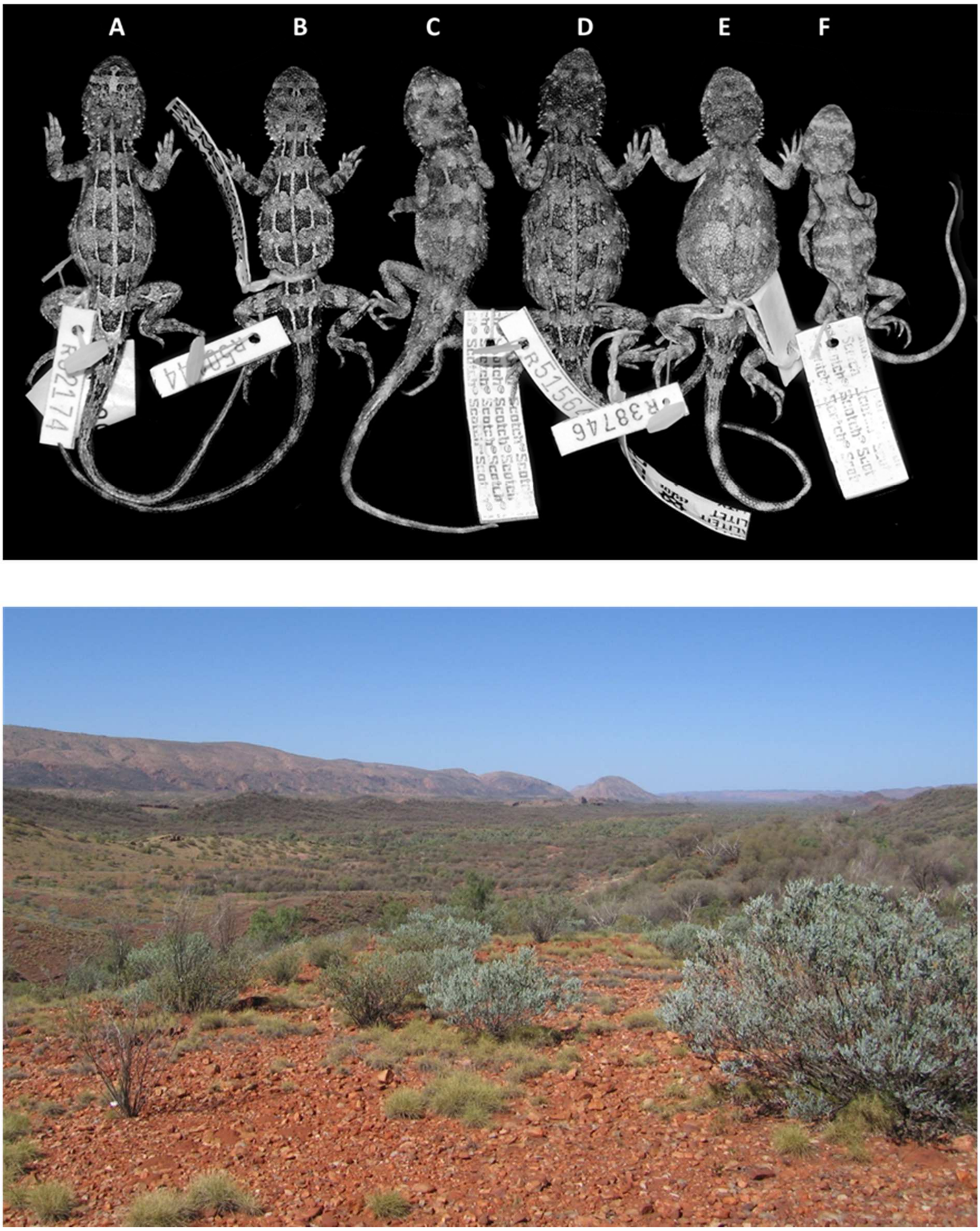

Figure S7. *Tympanocryptis centralis*: (a) variation within species (A, SAMA R62174, Morgan Range, Western Australia; B, SAMA R50144, Sentinel Hill, South Australia; C, SAMA R01559 C, Hermannsburg, Northern Territory; D, SAMA R51564, Amata, South Australia; E, SAMA R38746, Mount Cavenagh, Northern Territory; F, SAMA R 00318 B, MacDonnell Ranges, Northern Territory); and (b) habitat, West MacDonnell Ranges, NT (A. O'Grady).

(a)

(b)

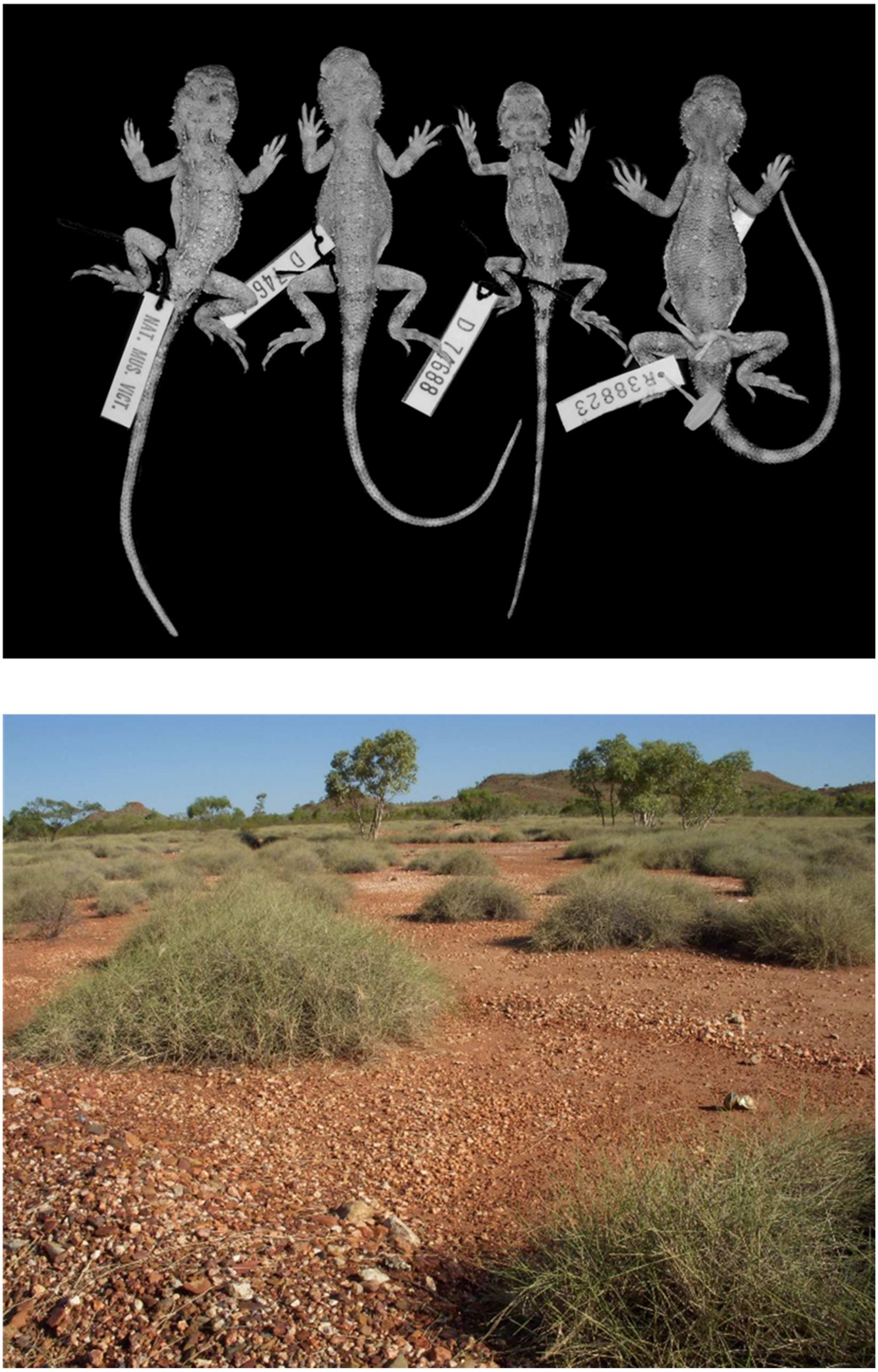

Figure S8. *Tympanocryptis rustica sp.nov.*: (a) variation within species (A, NMV D74684, male, Warrego Road, N of Tennant Creek; B, NMV D 74670, male, Warrego Road, N of Tennant Creek; C, NMV D74688, male, Warrego Road, N of Tennant Creek; D, SAMA R38823, female, Tennant Creek rubbish dump); and (b) habitat at type locality, Warrego Road, N of Tennant Creek (J. Melville).

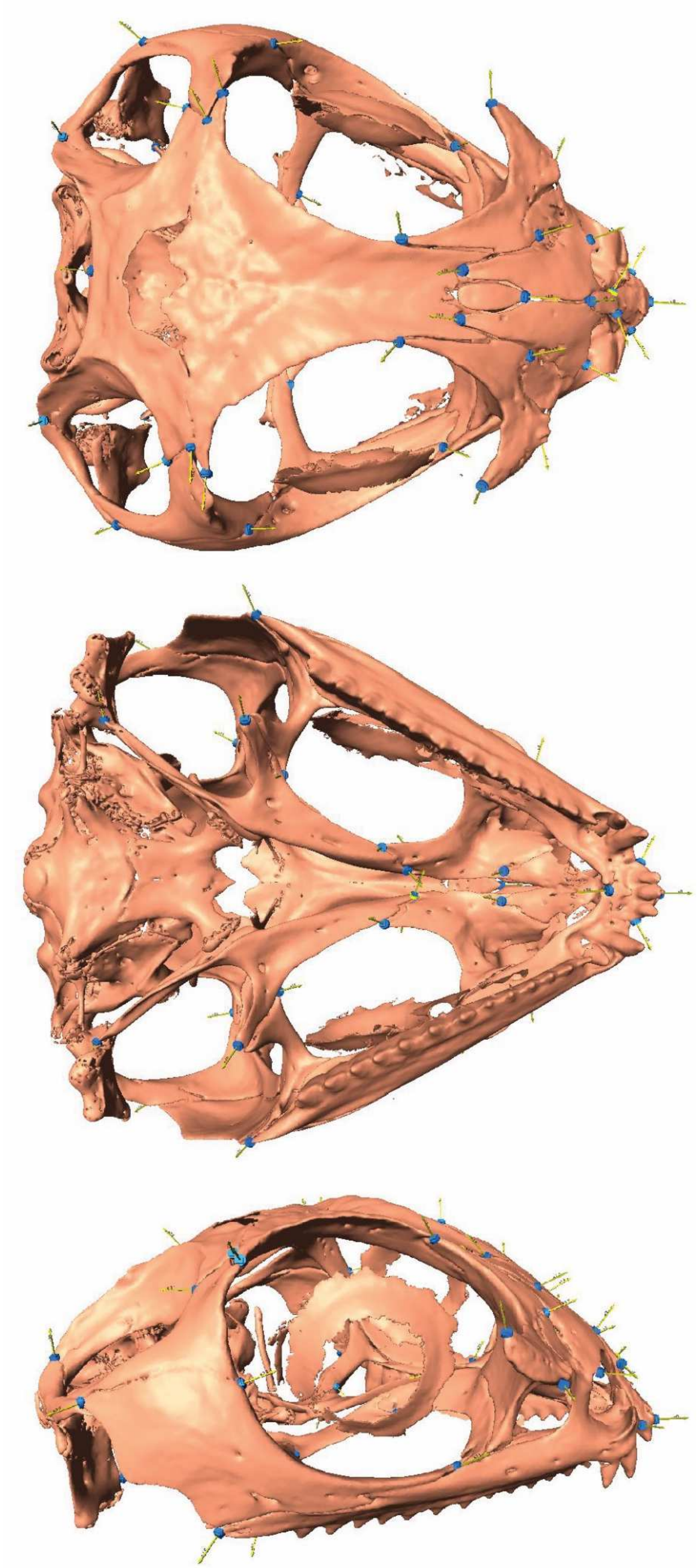

Figure S9. Locations of 49 landmarks placed on the cranial 3D models, shown on *Tympanocryptis pentalineata* (NMVD74073) in dorsal, ventral, and lateral view.
